# Impaired 1,25-dihydroxyvitamin D_3_ action underlies enthesopathy development in the *Hyp* mouse model of X-linked hypophosphatemia

**DOI:** 10.1172/jci.insight.163259

**Published:** 2023-09-08

**Authors:** Rakshya Rana, Jiana T. Baker, Melissa Sorsby, Supriya Jagga, Shreya Venkat, Shaza Almardini, Eva S. Liu

**Affiliations:** 1 Division of Endocrinology, Diabetes, and Hypertension, Brigham and Women’s Hospital, Boston, Massachusetts, USA.; 2 Harvard Medical School, Boston, Massachusetts, USA.

**Keywords:** Endocrinology, Bone disease

## Abstract

X-linked hypophosphatemia (XLH) is characterized by high serum fibroblast growth factor 23 (FGF23) levels, resulting in impaired 1,25-dihydroxyvitamin D_3_ (1,25D) production. Adults with XLH develop a painful mineralization of the tendon-bone attachment site (enthesis), called enthesopathy. Treatment of mice with XLH (*Hyp*) with 1,25D or an anti–FGF23 Ab, both of which increase 1,25D signaling, prevents enthesopathy. Therefore, we undertook studies to determine a role for impaired 1,25D action in enthesopathy development. Entheses from mice lacking vitamin D 1α-hydroxylase (*Cyp27b1*) (*C^–/–^*) had a similar enthesopathy to Hyp mice, whereas deletion of *Fgf23* in Hyp mice prevented enthesopathy, and deletion of both *Cyp27b1* and *Fgf23* in mice resulted in enthesopathy, demonstrating that the impaired 1,25D action due to high FGF23 levels underlies XLH enthesopathy development. Like *Hyp* mice, enthesopathy in *C^–/–^* mice was observed by P14 and was prevented, but not reversed, with 1,25D therapy. Deletion of the vitamin D receptor in scleraxis-expressing cells resulted in enthesopathy, indicating that 1,25D acted directly on enthesis cells to regulate enthesopathy development. These results show that 1,25D signaling was necessary for normal postnatal enthesis maturation and played a role in XLH enthesopathy development. Optimizing 1,25D replacement in pediatric patients with XLH is necessary to prevent enthesopathy.

## Graphical abstract

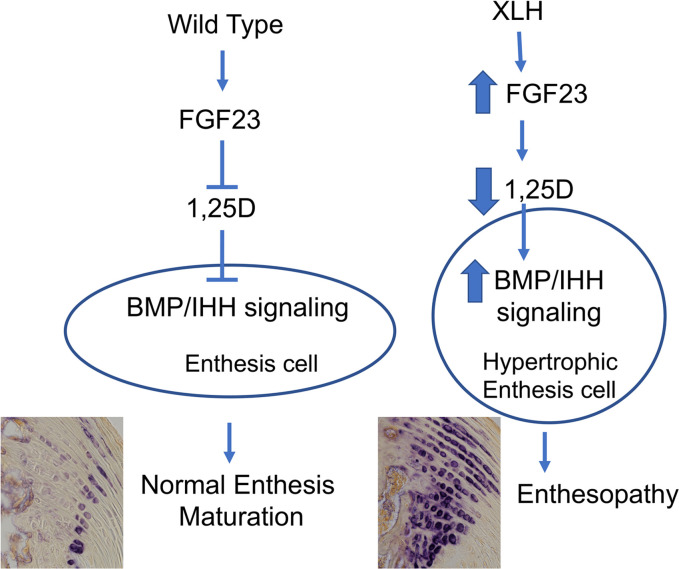

## Introduction

The enthesis is a specialized tissue that anchors the musculotendinous unit to bone. It functions to off-load the mechanical stress between muscle and bone and, therefore, is required for normal limb and body movement (1). The fibrocartilaginous enthesis is characterized by a gradual increase in mineral content through 4 zones consisting of tendon, unmineralized fibrocartilage, mineralized fibrocartilage, and bone (2). BMP and Indian hedgehog (IHH) signaling, both of which promote chondrogenesis, play essential roles in enthesis development by regulating enthesis fibrocartilage development (3, 4).

X-linked hypophosphatemia (XLH) is the most common form of inheritable rickets (1:20,000) that results from a mutation in the *PHEX* gene (5). It is characterized by increased circulating levels of FGF23, which leads to hypophosphatemia and suppression of vitamin D 1α-hydroxylase (*Cyp27b1*), resulting in decreased production of 1,25-dihydroxyvitamin D_3_ (1,25D) (6–8). The mineral ion and hormone alterations lead to growth retardation and poor skeletal mineralization (5). Up to 60% of adults with XLH develop enthesopathy, a debilitating condition that manifests as mineralization of the bone–tendon attachment site starting in the second decade of life (9). Enthesopathy leads to marked pain and impaired physical function and quality of life (5, 9–12); those affected by enthesopathy often require the use of ambulatory assist devices for movement (13). Common affected sites include the Achilles and patellar entheses, located in the ankle and knee, respectively. Our studies on enthesopathy in mice with XLH focus on the Achilles enthesis (14) because it is one of the most common sites for enthesopathy, seen in 74% of adults with XLH (10).

Similar to WT entheses, postnatal enthesis cells of *Hyp* mice originate from progenitors that express *Sox9* (a marker of chondrocytes) and scleraxis (*Scx*) (a marker of enthesis or tendon cells (14). Although both WT and *Hyp* enthesis cells have similar origins, further studies demonstrated that BMP and IHH signaling are increased in *Hyp* entheses, implicating these signaling pathways in XLH enthesopathy development (14). Treatment of *Hyp* mice with daily 1,25D or an Ab targeting FGF23 (FGF23Ab), which increases 1,25D levels, prevented enthesopathy in *Hyp* mice despite dramatic increases in *Fgf23* expression (14, 15). These results suggest impaired 1,25D signaling due to the elevated serum levels of FGF23 in XLH, not the direct effects of FGF23, plays a role in development of XLH enthesopathy.

To identify the relative roles of impaired 1,25D action and enhanced FGF23 action (both of which are due to high circulating levels of FGF23), and the *PHEX* mutation in enthesopathy development, we analyzed entheses from mice lacking *Cyp27b1*, *Fgf23*, or both; and from *Hyp* mice lacking *Cyp27b1*, *Fgf23*, or both. Mice lacking *Cyp27b1* or *Hyp* mice were treated with 1,25D starting early in postnatal development or after enthesopathy had already developed, to determine whether enhancing 1,25D action can prevent and/or attenuate BMP and IHH signaling in entheses. Furthermore, we examined entheses from mice lacking the vitamin D receptor (VDR) in Scx-expressing cells to determine if 1,25D acts directly on enthesis cells to suppress BMP and IHH signaling. In addition to determining a role for impaired 1,25D signaling due to enhanced FGF23 levels in XLH enthesopathy development, these studies will also define a role for 1,25D in normal postnatal enthesis maturation.

## Results

### Impaired 1,25D action in mice leads to enhanced BMP and IHH signaling in Achilles entheses.

Our previous studies demonstrated that Achilles entheses in *Hyp* mice have an expansion of hypertrophic-appearing fibrocartilage, or enthesopathy, cells (HECs) that stain positive for safranin O (SafO), a marker of cartilage proteoglycans, and alkaline phosphatase (ALP) activity, a marker of mineralization, at P30 (14). The SafO^+^ HECs have enhanced BMP and IHH signaling, both of which promote chondrogenesis (16–18). Increasing 1,25D action by treatment of *Hyp* mice with 1,25D or FGF23Ab prevents the increase in BMP and IHH and, thus, enthesopathy (14), despite increased *Fgf23* mRNA expression in bone and serum FGF23 levels (15). These results suggest that impaired 1,25D action due to increased circulating levels of FGF23 in *Hyp* mice, not increased FGF23-specific actions, enhances BMP and IHH signaling in entheses.

**Figure 1:**
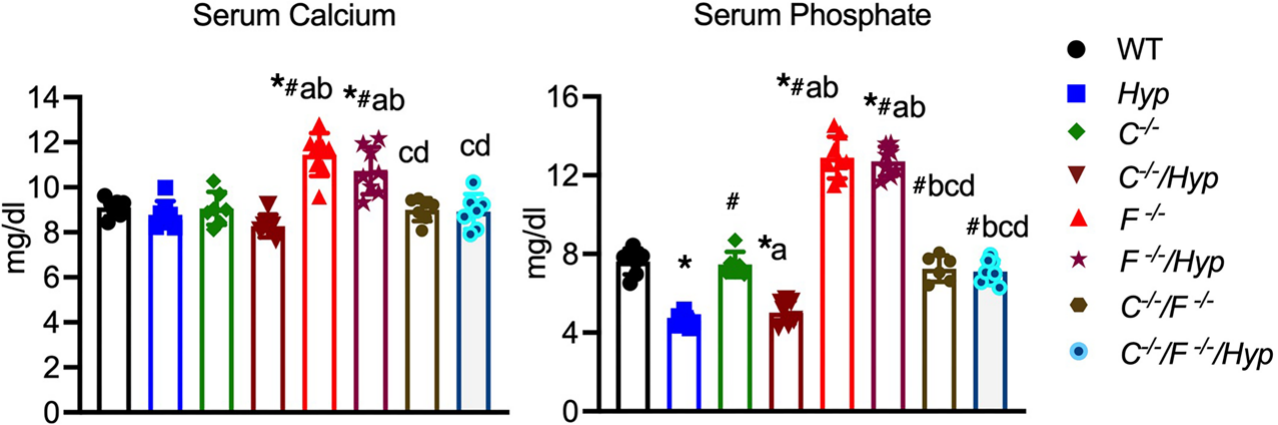
Serum mineral ion levels in mice lacking *Cyp27b1*, *Fgf23*, and/or *Phex*. Serum calcium and phosphate levels were measured for P30 mice lacking *Cyp27b1* (C^–/–^), *Fgf23* (*F^–/–^*), or both (*C^–/–^/F^–/–^*); *Hyp* mice lacking *Cyp27b1* (*C^–/–^/Hyp*), *Fgf23*(*F^–/–^/Hyp*), or both (*C^–/–^/F^–/–^/Hyp*); and WT and *Hyp* control mice. Data, presented as mean ± SD, are representative of at least 5 mice per age or genotype group. One-way ANOVA followed by Fisher’s least significant difference test was used to analyze significance between all genotype groups. *P < 0.05 versus WT; ^#^*P* < 0.05 versus *Hyp*; a indicates *P* < 0.05 versus *C^–/–^;* b indicates *P* < 0.05 versus *C^–/–^/Hyp*; c indicates *P* < 0.05 versus *F^–/–^*; and d indicates *P* < 0.05 versus *F^–/–^/Hyp*.

To elucidate the roles of 1,25D and FGF23 in regulating BMP and IHH signaling in entheses, Achilles entheses from mice lacking *Cyp27b1* (*C^–/–^*) (19) or *Fgf23* (*F^–/–^*) (7), as well as entheses from *Hyp* mice lacking *Cyp27b1* (*C^–/–^/Hyp*) or *Fgf23* (*F^–/–^/Hyp*) were analyzed. *C^–/–^* mice fed a high-calcium, high-phosphate rescue diet have normal serum calcium and phosphate levels; thus, analysis of *C^–/–^* entheses will enable determining whether impaired 1,25D signaling contributes to the enhanced BMP and IHH signaling observed in *Hyp* entheses and whether 1,25D action plays a role in normal enthesis maturation (Figure 1). *Hyp* mice lacking *Cyp27b1* have normocalcemia and hypophosphatemia; analyses of entheses from these mice will show if absence of 1,25D action in *Hyp* mice will worsen the *Hyp* enthesopathy phenotype. Previous characterization of the *F^–/–^* mice demonstrated that they have high serum 1,25D levels (7), leading to hypercalcemia and hyperphosphatemia (Figure 1). Examination of *F^–/–^* entheses will determine a role for FGF23 action in normal enthesis maturation, and analysis of entheses from *F^–/–^/Hyp* mice, which have high serum calcium and phosphate levels similar to that of *F^–/–^* mice (Figure 1) (7), will determine if the elevated circulating levels of FGF23 in *Hyp* mice contribute to the enhanced BMP and IHH signaling observed. Analysis of entheses in mice null for both *Cyp27b1* and *Fgf23* (*C^–/–^F^–/–^*), which have normal serum calcium and phosphate levels when fed a rescue diet, will differentiate whether impaired 1,25D production due to high FGF23 levels or actions specific to FGF23 lead to XLH enthesopathy. Because FGF23 overexpression is unlikely to be the only consequence of the PHEX mutation (20), ablation of both *Cyp27b1* and *Fgf23* in *Hyp* mice (*C^–/–^F^–/–^/Hyp*) will further elucidate if 1,25D- and FGF23-independent effects of the PHEX mutation influence XLH enthesopathy pathogenesis. Like *C^–/–^F^–/–^* mice, *C^–/–^F^–/–^/Hyp* mice weaned onto a rescue diet have normal serum calcium and phosphate levels (21) (Figure 1).

**Figure 2:**
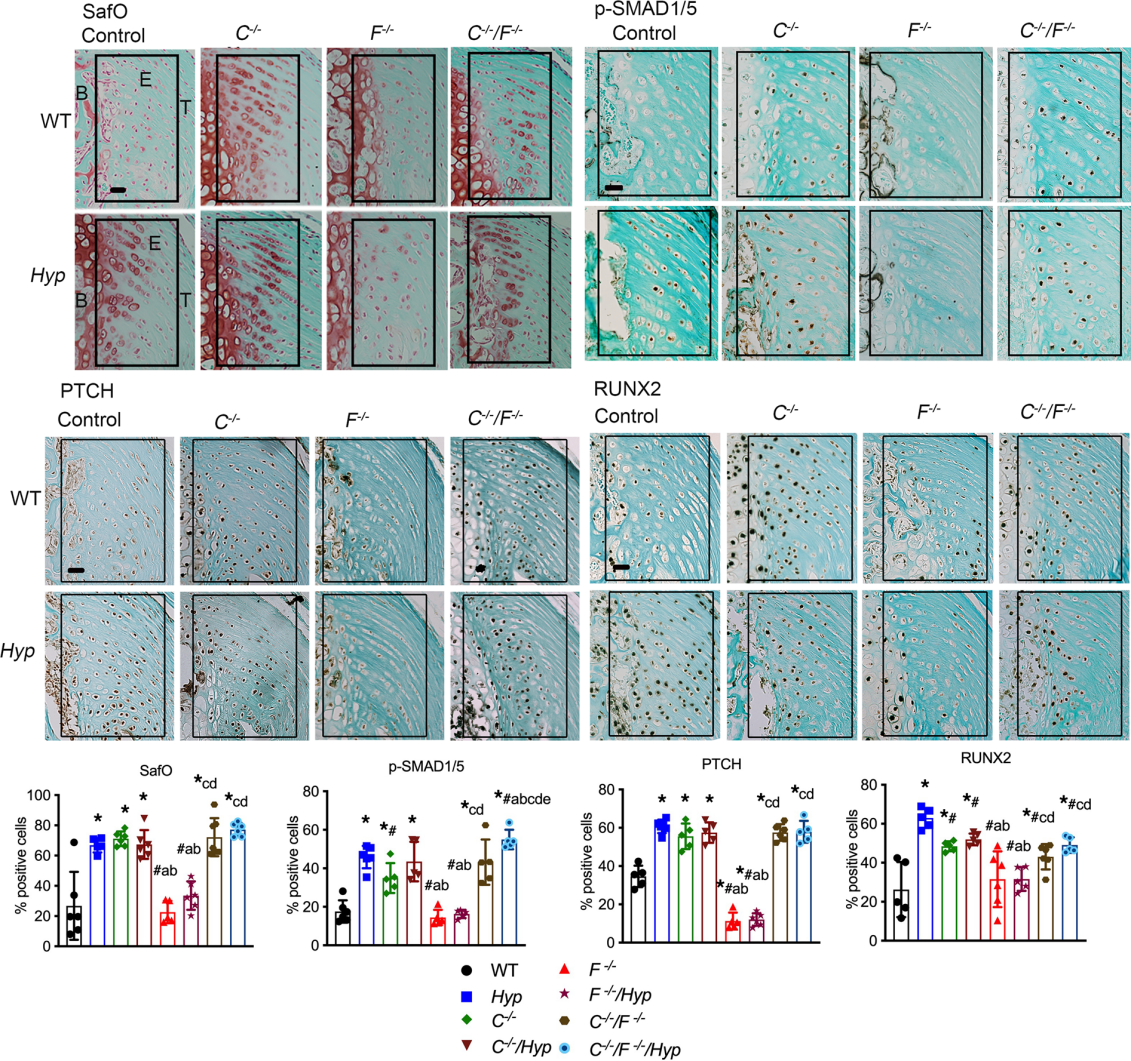
Entheses from all mice lacking *Cyp27b1* have increased BMP and IHH signaling. SafO staining and p-SMAD1/5/8, PTCH, and RUNX2 IHC were performed on entheses from P30 mice lacking *Cyp27b1* (*C^–/–^*), *Fgf23* (*F^–/–^*), or both (*C^–/–^/F^–/–^*); Hyp mice lacking *Cyp27b1* (*C^–/–^/Hyp*), *Fgf23* (*F^–/–^/Hyp*), or both (*C^–/–^/F^–/–^/Hyp*); and WT and *Hyp* control mice. The enthesis region is outlined with a black box. B, bone; E, enthesis; T, tendon. Quantitation of the percentage of SafO^+^ and the percentage of cells immunoreactive for p-SMAD1/5/8, PTCH, and RUNX2. Scale bar: 20 μm. Data, presented as mean ± SD, are representative of at least 5 mice per age or genotype group. The enthesis region is outlined with a black box. One-way ANOVA followed by Fisher’s least significant difference test was used to analyze significance between all genotype groups. *P < 0.05 versus WT; ^#^*P* < 0.05 versus *Hyp*; a indicates *P* < 0.05 versus *C^–/–^*; b indicates *P* < 0.05 versus *C^–/–^/Hyp*; c indicates *P* < 0.05 versus *F^–/–^*; and d indicates *P* < 0.05 versus *F^–/–^/Hyp*.

As previously reported, P30 *Hyp* Achilles entheses have increased cartilage proteoglycans, as shown by the expansion of SafO^+^ HECs adjacent to the secondary ossification center in the proximal enthesis region, whereas WT entheses have sparse SafO^+^ HECs in the distal portion of the enthesis (Figure 2) (14). BMP signaling promotes hypertrophy of chondrocytes by inducing phosphorylated SMAD1/5/8 (p-SMAD1/5/8) and regulates chondrocyte maturation by inducing IHH signaling (16–18). In support of the chondrogenic phenotype, the HECs in *Hyp* entheses have increased immunoreactivity for BMP target p-SMAD 1/5/8 and IHH targets Patched (PTCH) and RUNX2 (Figure 2), confirming that *Hyp* entheses have increased BMP and IHH signaling (14). Similar to *Hyp* control mice, P30 entheses from all groups of mice null for *Cyp27b1* (i.e., *C^–/–^, C^–/–^/Hyp, C^–/–^/F^–/–^, and C^–/–^/F^–/–^/Hyp*) have an expansion of SafO^+^ HECs with increased immunoreactivity for BMP target p-SMAD1/5/8 and IHH targets PTCH and RUNX2 (Figure 2). Deletion of *Cyp27b1* in mice, regardless if mice lack FGF23 and/or PHEX action, have an increase in the percentage of enthesis cells that stain positive for these BMP and IHH markers, with the percentage of SafO^+^, p-SMAD1/5^+^, and PTCH^+^ cells being similar to that in *Hyp* control entheses (Figure 2). The percentage of RUNX2^+^ cells is increased compared with WT but less than *Hyp* in all mice lacking *Cyp27b1*. *F^–/–^* mice resemble WT mice in that SafO^+^ cells that stain positive for BMP and IHH signaling markers are localized to the distal enthesis, where deletion of FGF23 leads to an even further decrease in PTCH immunoreactivity in the enthesis, as evidenced by the significant decrease in the percentage of PTCH^+^ cells in the *F^–/–^* entheses relative to WT (Figure 2). Ablation of *Fgf23* from *Hyp* mice prevents the increased SafO staining and immunoreactivity for p-SMAD1/5/8, PTCH, and RUNX2 observed in *Hyp* control entheses (Figure 2). Quantitation of the percentage of enthesis cells that stain positive for these BMP and IHH signaling markers confirms that F*^–/–^*/*Hyp* entheses have percentages of SafO^+^ and of p-SMAD1/5 and RUNX2 immunoreactive cells similar to those of WT entheses and an even further decrease in the percentage of PTCH^+^ cells compared with WT (Figure 2).

**Figure 3:**
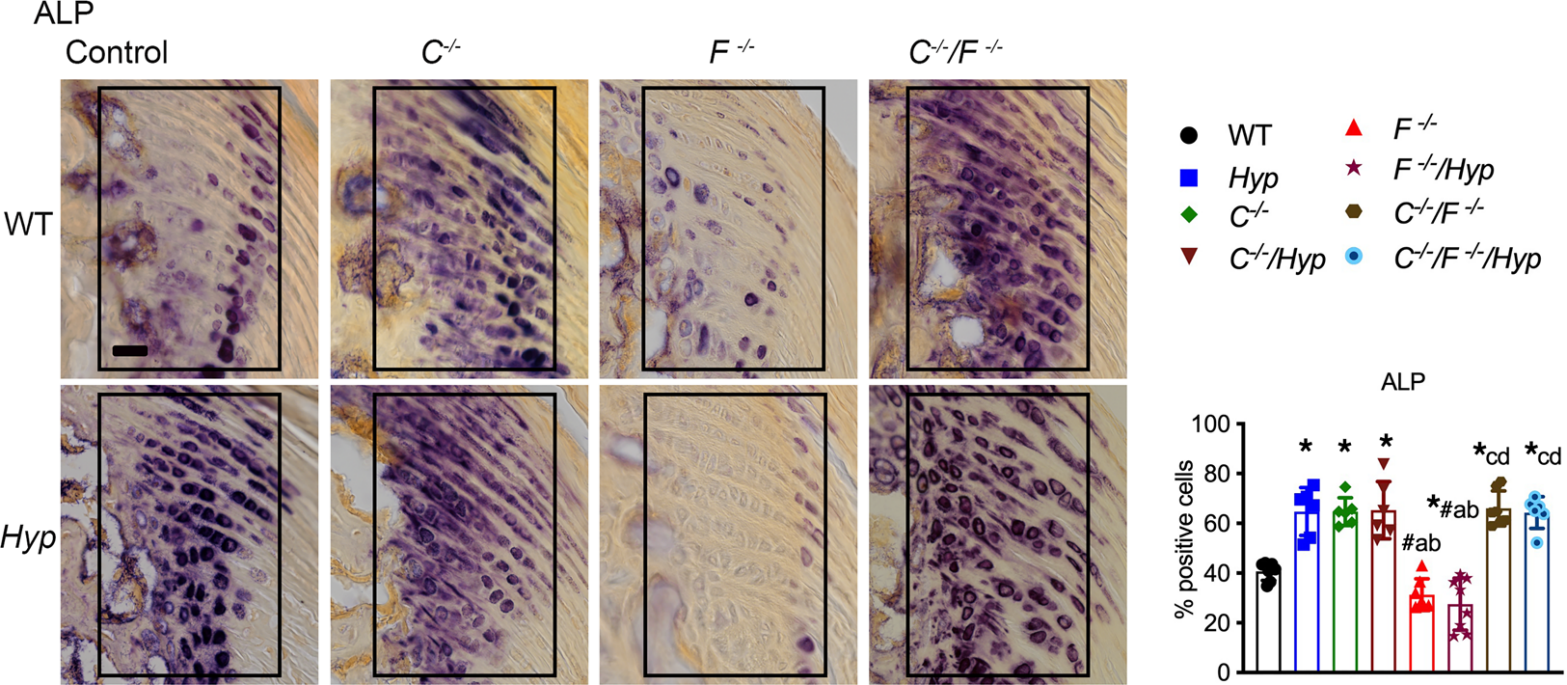
Entheses from all mice lacking *Cyp27b1* have increased ALP activity. Staining for ALP activity was performed on entheses from P30 mice lacking *Cyp27b1* (*C^–/–^*), *Fgf23* (*F^–/–^*), or both (*C^–/–^/F^–/–^*); *Hyp* mice lacking *Cyp27b1* (*C^–/–^/Hyp*), *Fgf23* (*F^–/–^/Hyp*), or both (*C^–/–^/F^–/–^/Hyp*); and WT and *Hyp* control mice. Quantitation of the percentage of ALP^+^ cells was performed. Scale bar: 20 μm. Data, presented as mean ± SD, are representative of at least 5 mice per age or genotype group. The enthesis region is outlined with a black box. One-way ANOVA followed by Fisher’s least significant difference test was used to analyze significance between all genotype groups. *P < 0.05 versus WT; ^#^*P* < 0.05 versus *Hyp*; a indicates *P* < 0.05 versus *C^–/–^*; b indicates *P* < 0.05 versus *C^–/–^/Hyp*; c indicates *P* < 0.05 versus *F^–/–^;* and d indicates *P* < 0.05 versus *F^–/–^/Hyp*.

ALP activity is expressed by hypertrophic chondrocytes (22) and required for matrix mineralization (23). Consistent with the enhanced BMP and IHH signaling observed in the entheses of mice lacking *Cyp27b*1, all mice null for *Cyp27b1* have an increased percentage of enthesis cells that stain positive for ALP (Figure 3). *F^–/–^* and *F^–/–^/Hyp* entheses do not have increased ALP activity; these entheses have similar percentages of ALP^+^ cells as WT (Figure 3). Taken together, these results support our hypothesis that the impaired 1,25D action due to increased FGF23 levels in *Hyp* mice contributes to the enhanced BMP and IHH signaling observed in *Hyp* mice and, thus, to enthesopathy development.

### Enhanced BMP and IHH signaling in Cyp27b1KO mice begins by P14.

**Figure 4:**
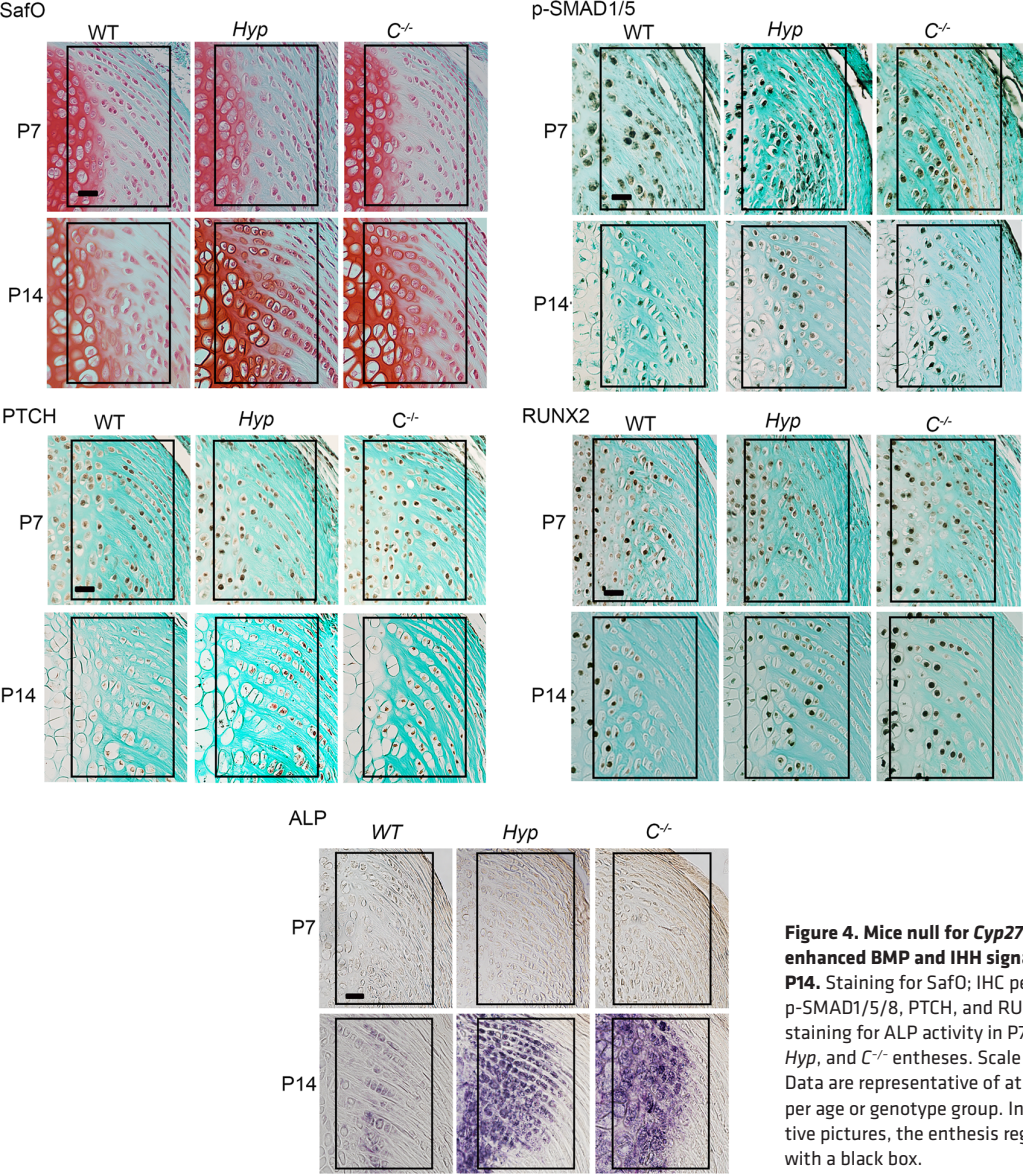
Mice null for *Cyp27b1* have enhanced BMP and IHH signaling by P14. Staining for SafO; IHC performed for p-SMAD1/5/8, PTCH, and RUNX2; and staining for ALP activity in P7 and P14 WT, *Hyp*, and *C^–/–^* entheses. Scale bar: 20 μm. Data are representative of at least 6 mice per age or genotype group. In all representative pictures, the enthesis region is outlined with a black box.

Our previous results demonstrated that *Hyp* entheses are normal at P7 but have increased BMP and IHH signaling by P14 (14). The results of the present study show that entheses from mice without 1,25D action have increases in BMP and IHH signaling and ALP activity similar to those of *Hyp* control mice (Figures 2 and 3). Therefore, to determine if entheses from mice lacking 1,25D action develop enhanced BMP and IHH signaling by 2 weeks of development, like *Hyp* control mice, entheses from P7 and P14 C*^–/–^* mice were examined. Like *Hyp* entheses, P7 *C^–/–^* entheses are not different from WT entheses in SafO staining; immunoreactivity for BMP and IHH markers p-SMAD1/5/8, PTCH, and RUNX2; or ALP activity (Figure 4). Similar to *Hyp* entheses, *C^–/–^* entheses develop an expansion of SafO^+^, ALP^+^ cells that are immunoreactive for p-SMAD1/5/8, PTCH, and RUNX2 by P14, thus demonstrating that enthesopathy in both *Hyp* and *C^–/–^* mice develops early in the postnatal period. At P30 and P60, BMP and IHH signaling and ALP activity remain elevated in *Hyp* and *C^–/–^* entheses, with staining for these markers being decreased in P60 entheses compared with P30 entheses (Supplemental Figure 1; supplemental material available online with this article; https://doi.org/10.1172/jci.insight.163259DS1).

**Figure 5:**
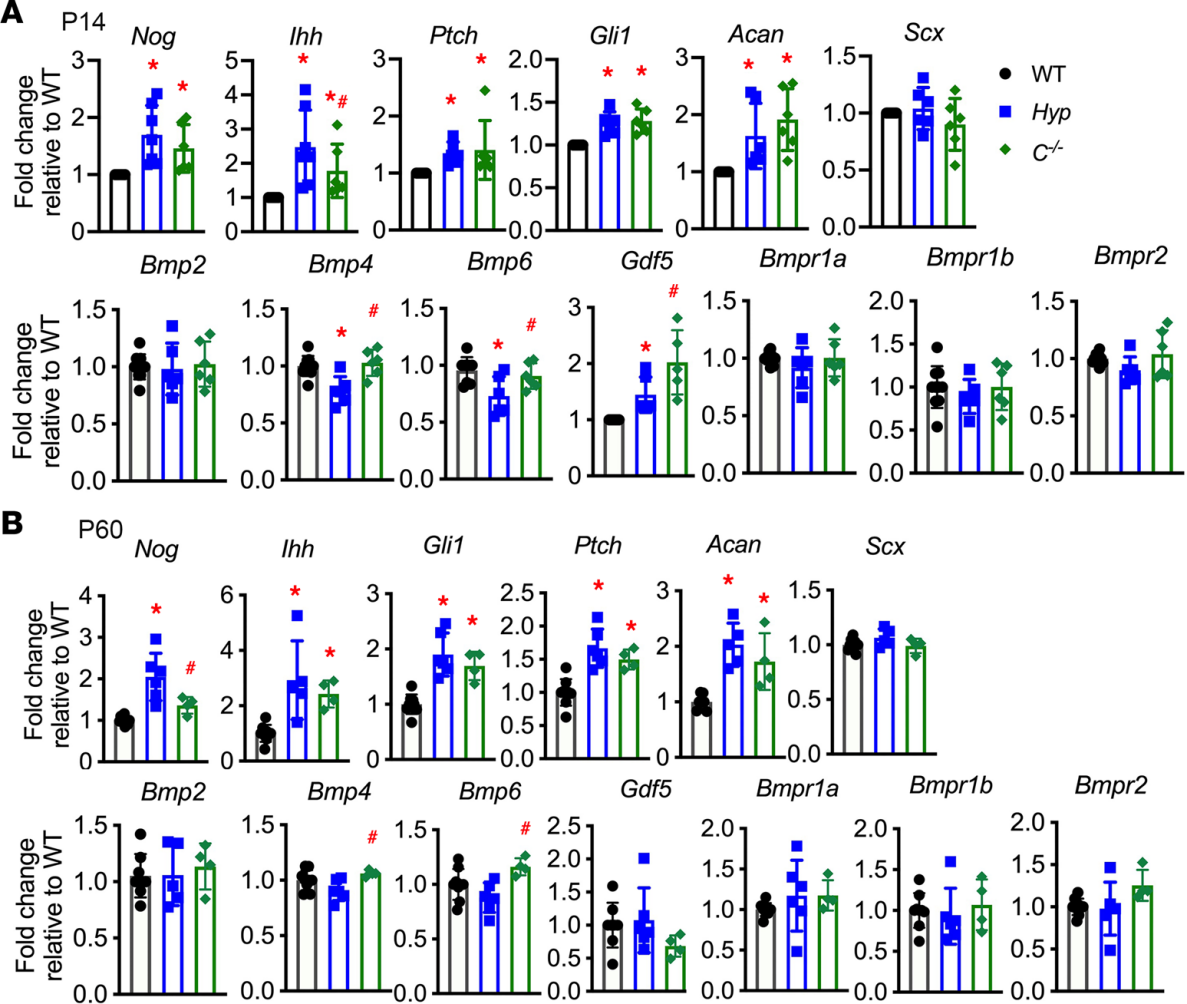
Mice null for Cyp27b1 have enhanced mRNA expression of BMP and IHH target genes. (**A** and **B**) mRNA expression of BMP and IHH signaling target genes, *Acan*, and *Scx*, in P14 and P60 WT, *Hyp*, and *C^–/–^* entheses. Gene expression is normalized to that of WT. Data, presented as mean ± SD, are representative of at least 6 mice per genotype in P14 entheses and 4–6 entheses per genotype in P60 entheses. One-way ANOVA followed by Fisher’s least significant difference test was used to analyze significance between all genotype groups. *P < 0.05 versus WT; ^#^*P* < 0.05 versus *Hyp*.

Consistent with increased immunoreactivity for BMP and IHH signaling markers seen in *Hyp* entheses, there is also a significant increase in mRNA expression of BMP and IHH target genes in *Hyp* entheses by P14 (14) (Figure 5A). P14 entheses from *C^–/–^* mice demonstrate increased expression of BMP target genes *Noggin* (*Nog*) and *Ihh*, with *Ihh* expression being slightly, but significantly, higher in *Hyp* entheses as compared with *C^–/–^* entheses. P14 *C^–/–^* entheses also have enhanced expression of IHH target genes *Ptch1*, *Runx2*, and *Gli1*, and cartilage proteoglycan marker aggrecan (*Acan*), whereby the fold increase in expression of these genes relative to WT entheses was similar to that seen in *Hyp* entheses (Figure 5). Gene expression of these BMP and IHH signaling markers remains elevated in P60 *Hyp* and *C^–/–^* entheses (Figure 5B).

BMP family members, including BMP2, BMP4, BMP6, and GDF5, are important for chondrogenesis. They bind and activate a multimeric complex that includes type I and type II BMP receptors (BMPRs), which leads to the phosphorylation of SMAD1/5/8 (24). BMP2 and BMP6 have been implicated in murine ankylosing spondylosis enthesitis development, and BMP4 is required for the formation of the bony ridge onto which the deltoid tendon inserts (4, 25). Also, lineage tracing studies have demonstrated that enthesis cells descend from *Gdf5*-expressing progenitors (26), and studies have demonstrated that GDF5 promotes chondrocyte hypertrophy (27). BMP family members bind different BMPRs with different affinities: BMP2 and BMP4 preferentially bind to the BMPR2/BMPR1A complex, and GDF5 induces BMP signaling by preferentially activating the BMPR2/BMPR1B complex (24, 28–30).

In P14 entheses, mRNA expression of *Gdf5* was increased in both *Hyp* and *C^–/–^* entheses, whereas *Bmp2*, *Bmp4*, and *Bmp6* mRNA expression was not elevated in either *Hyp* or *C^–/–^* entheses (Figure 5A), suggesting enhanced *Gdf5* expression in *Hyp* entheses contributes to the enhanced BMP and IHH signaling observed in *Hyp* and *C^–/–^* entheses. By P60, *Bmp2*, *Bmp4*, *Bmp6*, and *Gdf5* expression is normal in *Hyp* and *C^–/–^* entheses (Figure 5B). Consistent with the decreased immunoreactivity for BMP and IHH signaling markers in P60 versus P14 entheses of WT, *Hyp*, and *C^–/–^* mice, mRNA expression of IHH-signaling target genes *Ihh,* *Gli1,* and *Acan* is also decreased in P60 compared with P14 entheses (Supplemental Figure 2). Compared with P14 entheses, P60 entheses have lower mRNA expression of *Gdf5*, higher expression of *Bmp2,* and similar expression of *Bmp4* and *Bmp6* (Supplemental Figure 2). These results indicate that expression of BMP and IHH markers decreases with age. In both P14 and P60 entheses, expression of *Bmpr1a*, *Bmpr1b*, and *Bmpr2* remains similar to WT in *Hyp* and *C^–/–^* entheses (Figure 5), though expression of *Bmpr1b* is decreased in P60 entheses, whereas *Bmpr1a* and *Bmpr2* expression is unchanged in P60 entheses compared with that of P14 (Supplemental Figure 2). The mRNA expression of *Scx*, a marker of tendon and entheses, was similar in P14 and P60 WT, *Hyp*, and *C^–/–^* entheses, indicating that all entheses were consistently isolated (Figure 5).

### Systemic 1,25D therapy prevents enthesopathy in Cyp27b1KO mice.

**Figure 6:**
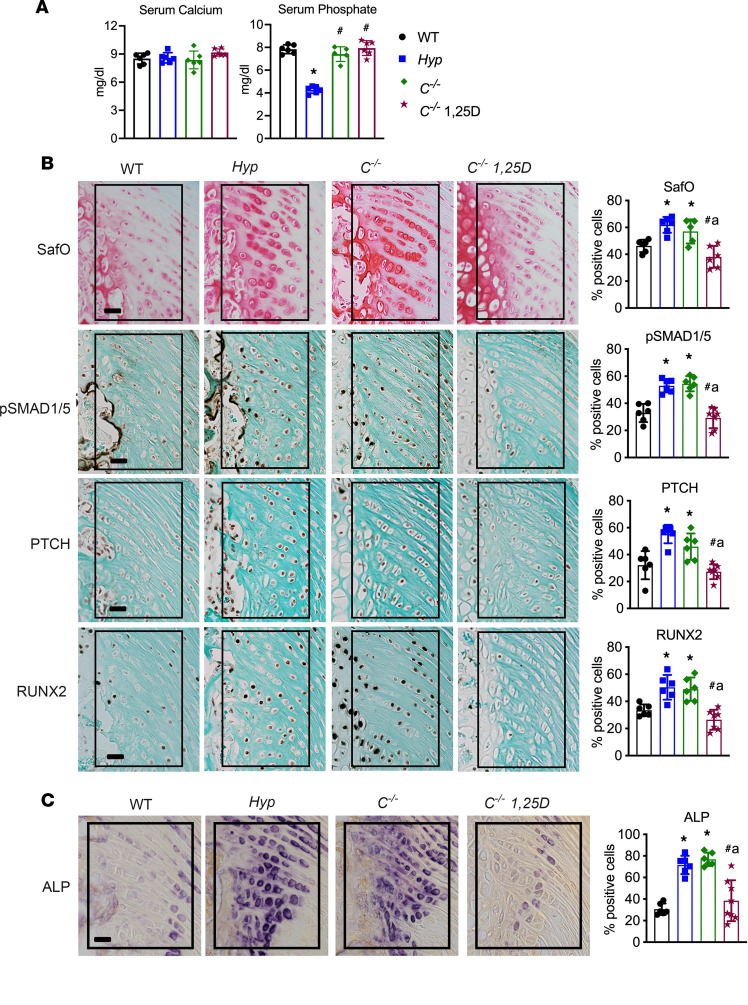
Treatment of *C^–/–^* mice with 1,25D starting on P2 prevents enthesopathy. (**A**) Serum calcium and phosphate levels are measured in *C^–/–^* mice treated with 1,25D on P2 to P30 as well as P30 WT, *Hyp*, and *C^–/–^* control mice. (**B**) SafO staining and p-SMAD1/5/8, PTCH, and RUNX2 IHC were performed on P30 entheses from WT, *Hyp*, and *C^–/–^* control mice and *C^–/–^* mice treated with 1,25D from P2 to P30. The percentage of cells positive for staining was quantitated. (**C**) Staining for ALP activity was performed on P30 entheses, with quantitation of the percentage of positive ALP enthesis cells. In all representative pictures, the enthesis region is outlined with a black box. Scale bar: 20 μm. Data, presented as mean ± SD, are representative of 5–7 mice per age or genotype group. One-way ANOVA followed by Fisher’s least significant difference test was used to analyze significance between all genotype groups. *P < 0.05 versus WT; ^#^*P* < 0.05 versus *Hyp*; a indicates *P* < 0.05 versus *C^–/–^*.

Our previous studies demonstrated that treatment of *Hyp* mice with daily 1,25D (175 pg/g/d) from P2 to P30 increased serum phosphate levels, maintained normocalcemia, and prevented the increase in cartilage proteoglycans, BMP and IHH signaling, and ALP activity in entheses, thus blocking the development of enthesopathy in *Hyp* mice (14). To determine if restoration of 1,25D signaling prior to enthesopathy development similarly prevents enthesopathy in *C^–/–^* mice, these mice were treated with daily 1,25D from P2 to 30, with *C^–/–^* mice receiving 175 pg/g/d 1,25D from P2 to P18 and then 80 pg/g/d 1,25D from P18 to P30 to prevent hypercalcemia in the setting of being weaned onto the high-calcium rescue diet. The *C^–/–^* control and treated mice maintained normal serum calcium and phosphate levels while being maintained on this diet (Figure 6A). Like Hyp mice, treatment of *C^–/–^* mice with 1,25D starting on P2 decreased SafO staining, immunoreactivity for BMP and IHH target genes, and ALP activity relative to *C^–/–^* and *Hyp* control mice (Figure 6, B and C). Quantitation of the percentage of cells staining positive for these parameters in treated *C^–/–^* mice was the same as in WT mice (Figure 6, B and C), thus showing that restoring 1,25D action in *C^–/–^* mice early in postnatal development prevents enthesopathy.

### 1,25D acts directly on Scx^+^ cells to suppress BMP and IHH signaling.

**Figure 7:**
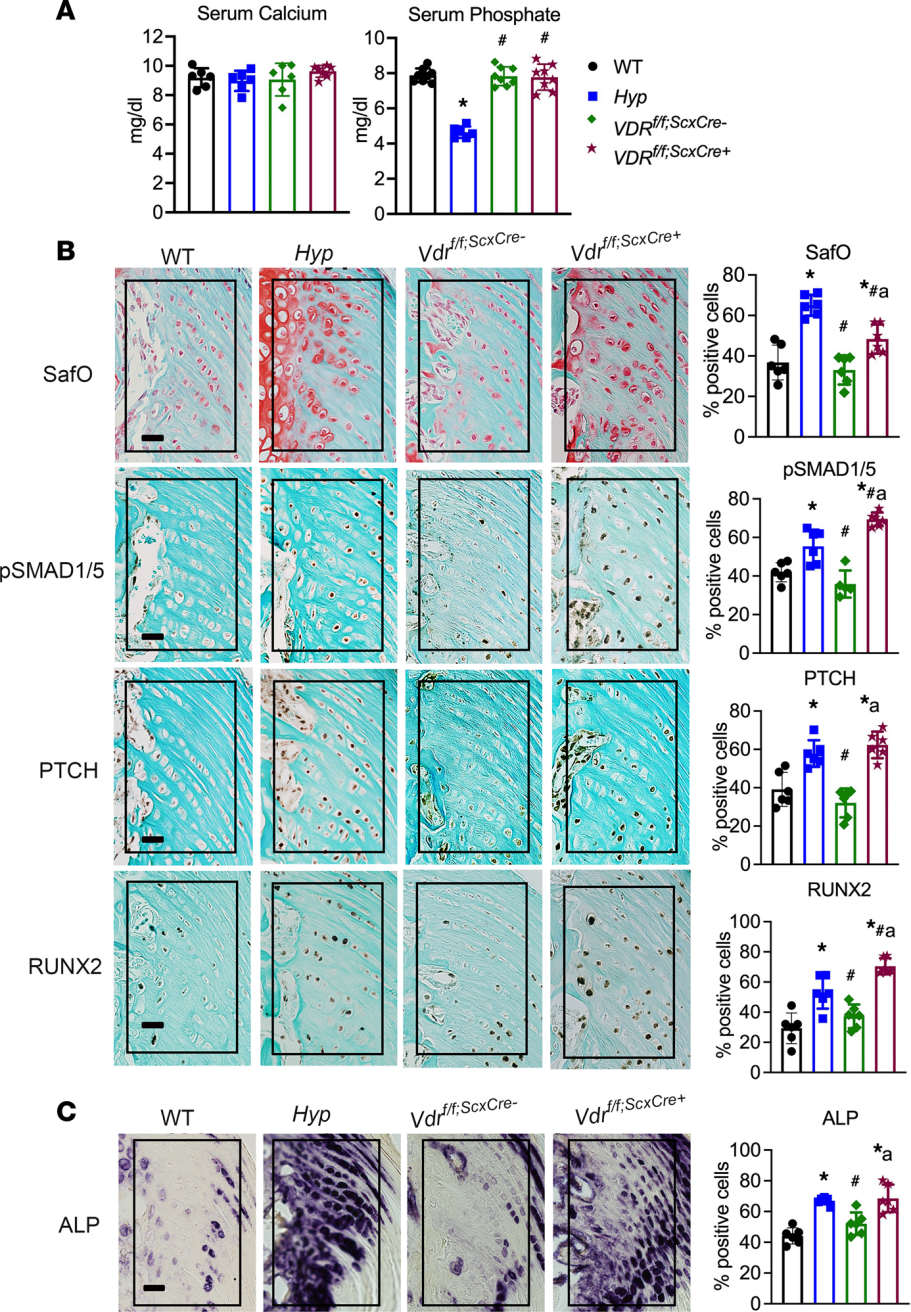
Deletion of the *Vdr* in Scx+ cells leads to enhanced BMP and IHH signaling in entheses. (**A**) Serum calcium and phosphate levels were measured in *Vdr^f/f;ScxCre+^* mice and controls (WT; *Hyp*, *Vdr^f/f;ScxCre–^* mice). (**B**) SafO staining and p-SMAD1/5/8, PTCH, and RUNX2 IHC were performed on P30 entheses from *Vdr^f/f;ScxCre+^* mice and controls (WT; *Hyp*, *Vdr^f/f;ScxCre–^* mice). (**C**) Staining for ALP activity was performed on P30 entheses. In all representative pictures, the enthesis region is outlined with a black box. Scale bar: 20 μm. Data, presented as mean ± SD, are representative of 6–8 mice per age or genotype group. One-way ANOVA followed by Fisher’s least significant difference test was used to analyze significance between all genotype groups. *P < 0.05 versus WT; ^#^*P* < 0.05 versus *Hyp*; a indicates *P* < 0.05 versus *VDR^f/f;ScxCre–^*.

Our data demonstrate that entheses from mice lacking 1,25D action have increased BMP and IHH signaling early in development, by P14, and that restoration of 1,25D action with systemic 1,25D therapy prevents enthesopathy development. To determine if 1,25D acts directly on enthesis cells to regulate BMP and IHH signaling, *Vdr* was deleted in cells expressing *Scx* by mating mice with floxed *Vdr* alleles (31) to mice expressing Cre recombinase under the direction of the *Scx* promoter (4) (*Vdr^f/f;ScxCre+^*). *Vdr^f/f;ScxCre+^* mice and corresponding *Vdr^f/f;ScxCre–^* control mice have normal serum calcium and phosphate levels (Figure 7A). *Vdr^f/f;ScxCre–^* control entheses had similar staining and percentages of positive cells for SafO, BMP and IHH signaling markers, and ALP activity as WT entheses. Lack of 1,25D signaling in Scx^+^ cells resulted in an enthesopathy similar to that in *Hyp* control mice and that observed in *C^–/–^* mice (Figure 7, B and C). *Vdr^f/f;ScxCre+^* entheses have an expansion of SafO^+^ HECs with increased immunoreactivity for BMP and IHH signaling markers p-SMAD1/5/8, PTCH, and RUNX2, and increased ALP activity (Figure 7, B and C), as confirmed by quantitation of the percentage of positive cells for these markers, thereby demonstrating that 1,25D acts directly on Scx^+^ cells to suppress BMP and IHH signaling and enthesopathy development.

### 1,25D suppresses GDF5-induced BMP and IHH signaling in chondrogenic cells, and VDR expression is decreased in Hyp entheses.

**Figure 8:**
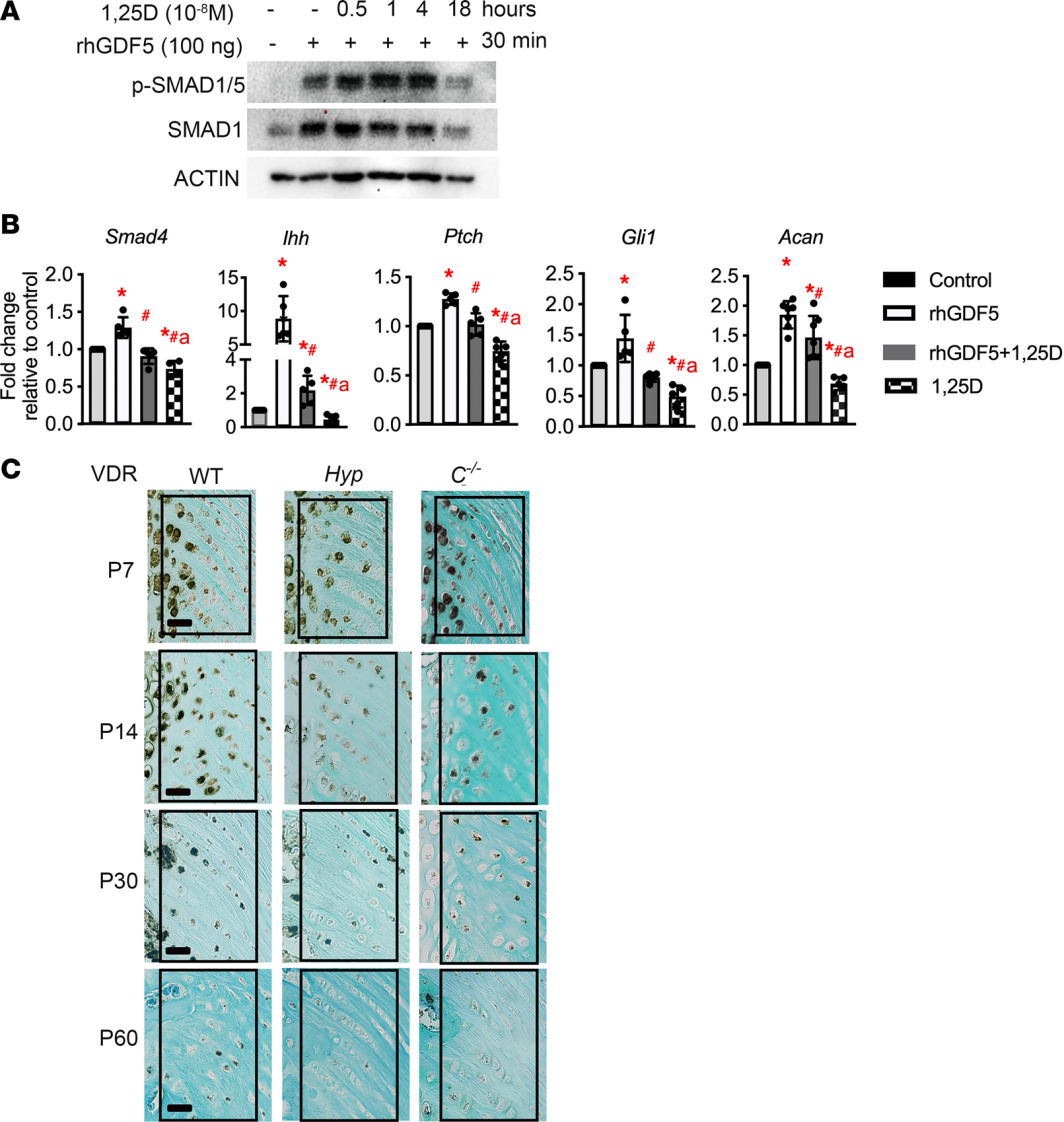
1,25D blocks rhGDF5 induced expression of BMP and IHH genes and p-SMAD1/5 and VDR expression is decreased in Hyp entheses by P14. (**A**) Primary murine chondrocytes were pretreated for 0.5, 1, 4, or 18 hours with or without (–) 1 × 10^–8^ M 1,25D prior to incubation with (+) or without (–) 100 ng/mL rhGDF5 (for 30 minutes) and subjected to Western blot analyses for p-SMAD1/5/9, SMAD1, and β-actin. Data are representative of those obtained from 3 independent experiments. (**B**) Primary murine chondrocytes were pretreated for 18 hours with 1 × 10^–8^ M 1,25D followed by treatment with 200 ng/mL rhGDF5 (for 4 hours). Gene expression analyses were performed for BMP and IHH target genes. Data are representative of those obtained from 5–7 independent experiments. One-way ANOVA followed by Fisher’s least significant difference test was used to analyze significance between all genotype groups. *P < 0.05 versus WT; ^#^*P* < 0.05 versus rhGDF5; a indicates *P* < 0.05 versus rhGDF5 plus 1,25D. (**C**) IHC for VDR was performed on P7, P14, P30, and P60 entheses from WT, *Hyp*, and *C^–/–^* mice. In all representative pictures, the enthesis region is outlined with a black box. Scale bar: 20 μm. Data are representative of 6 mice per age or genotype group.

Because impaired 1,25D action leads to enhanced BMP and IHH signaling in entheses (Figures 2 and 7), it was hypothesized that 1,25D directly decreases BMP and IHH signaling. Enthesis cells share features with chondrocytes, and *Gdf5* expression is increased in entheses of *Hyp* mice and *C^–/–^* mice. Thus, to determine if 1,25D directly blocks BMP signaling, primary murine chondrocytes were pretreated with increasing durations of 1 × 10^–8^ M 1,25D and then incubated with rhGDF5. 1,25D blocked GDF5-induced p-SMAD1/5 and expression of SMAD1 with 18 hours of pretreatment with 1,25D (Figure 8A). SMAD4 forms heterotrimers with SMAD1, 5, or 8/9 to enable translocation of these factors into the nucleus and regulation of target gene expression (24), and BMP signaling activates IHH signaling in chondrocytes (17, 18). Therefore, to determine the effect of 1,25D on expression of these regulators of BMP and IHH signaling, primary chondrocytes were pretreated 1 × 10^–8^ M 1,25D followed by rhGDF5. 1,25D decreased GDF5-induced expression of *Smad4*, IHH signaling target genes (*Ihh*, *Ptch,* *Gli1*), and of the chondrogenic marker *Acan* (Figure 8B).

To determine if altered VDR expression plays a role in the impaired 1,25D action in entheses of Hyp mice, IHC for the VDR was performed (Figure 8C). At P7, staining for the VDR is normal in Hyp, entheses. By P14, *Hyp* entheses have a decrease in immunoreactivity for the VDR compared with WT entheses, suggesting that a decrease in VDR expression in *Hyp* entheses contributes to the impaired 1,25D action and, thus, enthesopathy development. There is also a gradual decrease in expression of the VDR in entheses with increasing age, because immunoreactivity for the VDR in WT and *Hyp*, and *C^–/–^* entheses progressively decrease from P14 to P60 entheses regardless of genotype.

### Systemic 1,25D therapy cannot reverse enthesopathy in Hyp and Cyp27b1KO mice.

**Figure 9:**
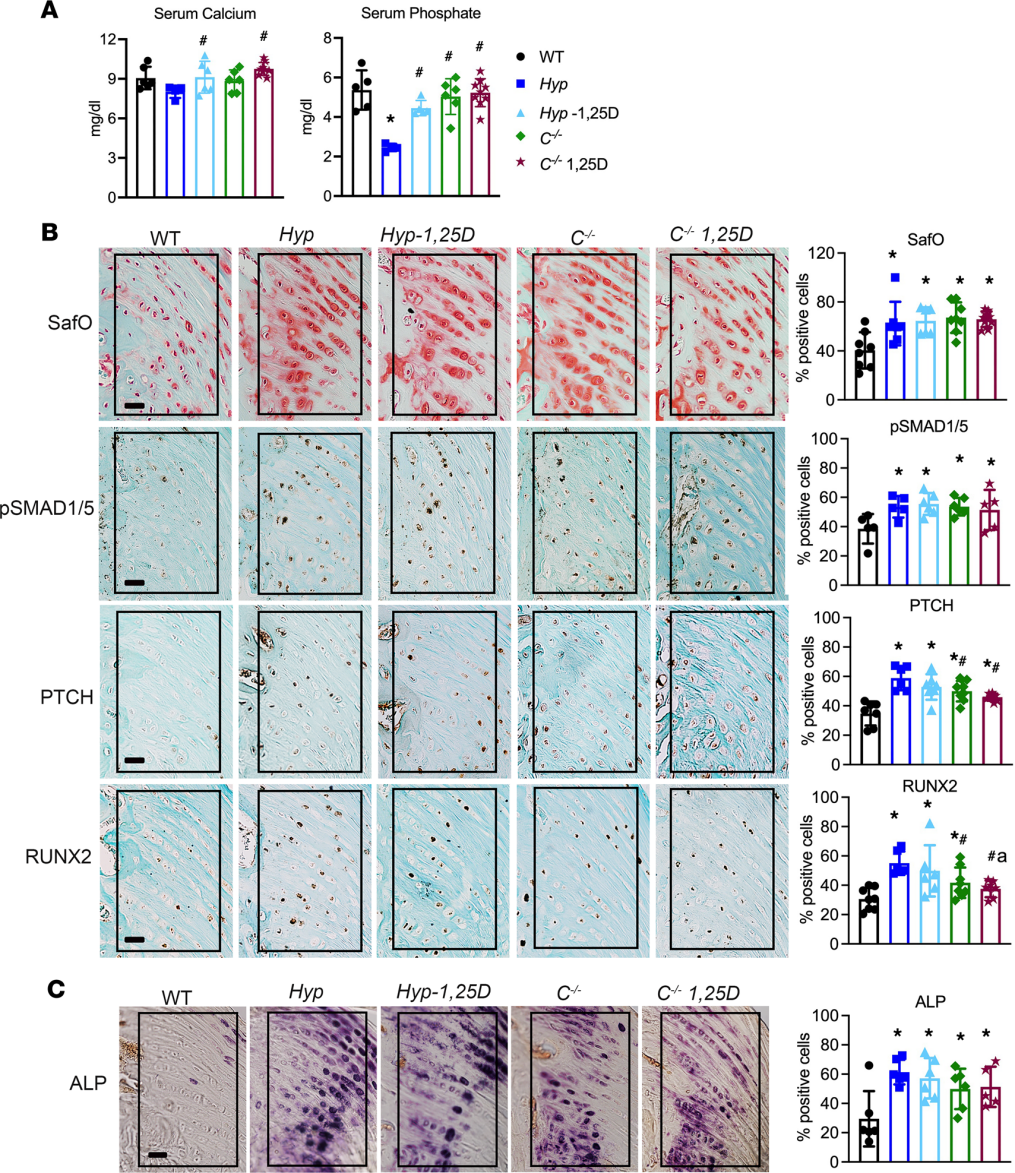
Treatment of *Hyp* and *C^–/–^* mice with 1,25D starting on P30 does not reverse enthesopathy. (**A**) Serum calcium and phosphate levels were measured in *Hyp* and *C^–/–^* mice treated with 1,25D from P30 to P60 and P60 WT, *Hyp*, and *C^–/–^* control mice. (**B**) Staining for SafO and IHC for p-SMAD1/5/8, PTCH, and RUNX2 were performed on entheses from P60 WT, *Hyp*, and *C^–/–^* control mice and *Hyp* and *C^–/–^* mice treated from P30 to P60 with daily 1,25D. The percentages of cells positive for SafO and for BMP and IHH signaling markers were quantitated. (**C**) Staining for ALP activity was performed on P60 entheses and the percentage of positive cells was quantitated. In all representative pictures, the enthesis region is outlined with a black box. Scale bar: 20 μm. Data, presented as mean ± SD, are representative of 6–8 mice per age or genotype group. One-way ANOVA followed by Fisher’s least significant difference test was used to analyze significance between all genotype groups. *P < 0.05 versus WT; ^#^*P* < 0.05 versus *Hyp*; a indicates *P* < 0.05 versus *C^–/–^*.

Because 1,25D therapy in *Hyp* and *C^–/–^* mice starting at P2 prevents enthesopathy, *Hyp* and *C^–/–^* mice were treated with daily 1,25D starting at P30 to determine if restoring 1,25D signaling after enthesopathy has developed can reverse enthesopathy. *Hyp* mice were treated with 175 pg/g/d 1,25D from P30 to P60, and *C^–/–^* mice were treated with a lower dose of 80 pg/g/d 1,25D from P30 to P60 to maintain normocalcemia while being fed the high-calcium, high-phosphate rescue diet. On P60, treated *Hyp* and *C^–/–^* mice and *Hyp* and *C^–/–^* control mice have normal serum calcium levels. Treatment of Hyp mice with 1,25D starting on P30 resulted in normalized serum phosphate levels and maintained normal levels in *C^–/–^* mice (Figure 9A). P60 entheses from *Hyp* and *C^–/–^* mice receiving 1,25D therapy starting on P30 continue to exhibit an expansion of SafO^+^ cells with enhanced immunoreactivity for p-SMAD1/5/8, PTCH, and RUNX2, and enhanced ALP activity similar to that observed in untreated *Hyp* and *C^–/–^* control mice (Figure 9, B and C). By P60, quantitation of the percentage of PTCH^+^ and RUNX2^+^ cells in *C^–/–^* control and *C^–/–^* treated entheses is mildly decreased relative to that of Hyp controls (Figure 9B). However, 1,25D treatment does not alter *C^–/–^* staining for these IHH signaling markers in *C^–/–^* mice. These data demonstrate that restoring 1,25D signaling after enthesopathy has developed does not attenuate BMP and IHH signaling in *Hyp* and *C^–/–^* entheses.

### Loss of the VDR in Scx^+^ cells starting on P30 does not result in enthesopathy.

**Figure 10:**
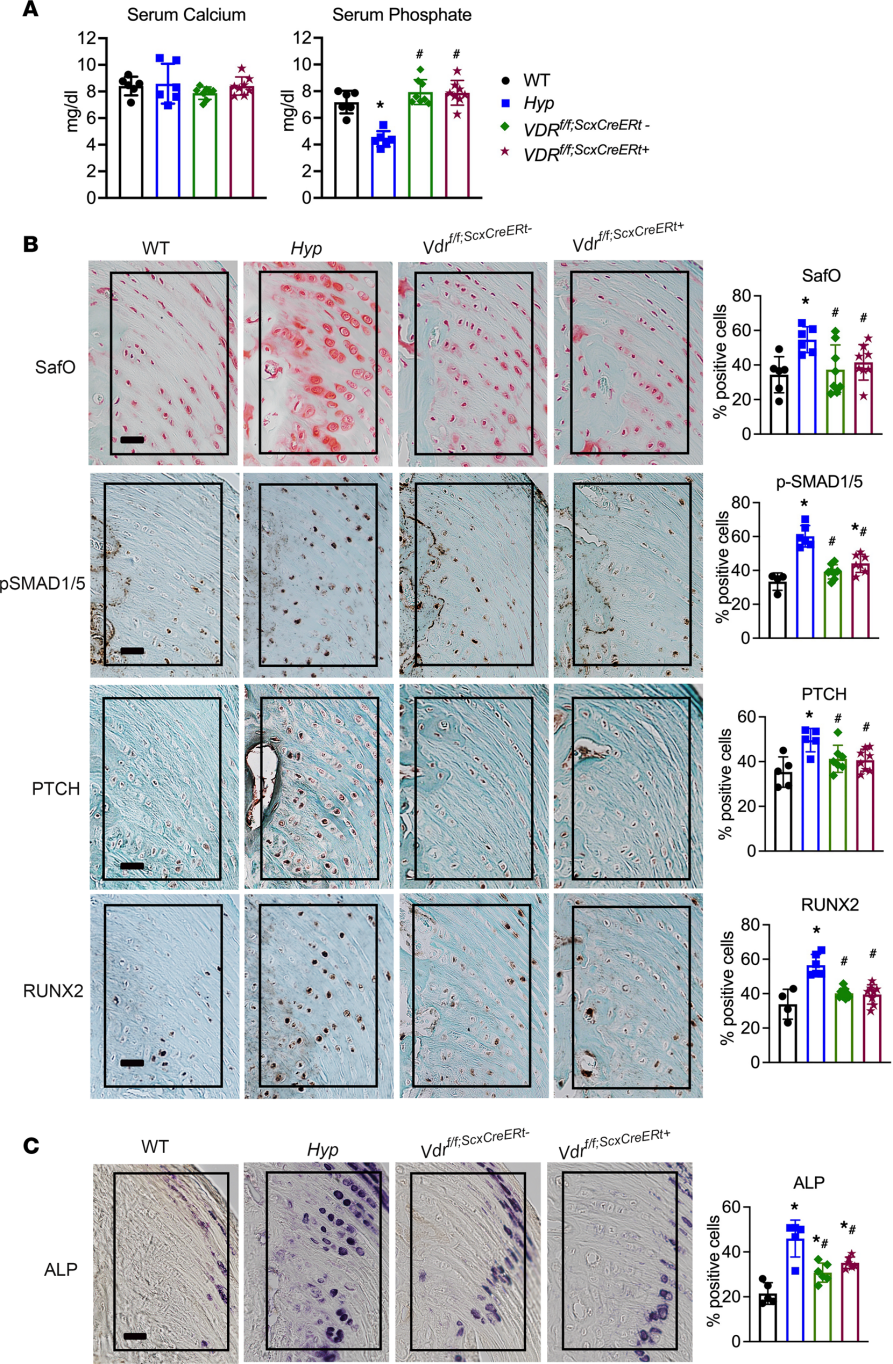
Deletion of the *Vdr* starting on P30 does not increase BMP and IHH signaling in entheses. (**A**) Serum calcium and phosphate levels were measured in *Vdr^f/f;ScxCreERt+^* mice and controls (WT; *Hyp*, *Vdr^f/f;ScxCreERt–^* mice). (**B**) SafO staining and p-SMAD1/5/8, PTCH, and RUNX2 IHC were performed on P60 entheses from *Vdr^f/f;ScxCreERt+^* mice and controls (WT; *Hyp*, *Vdr^f/f;ScxCreERt–^* mice). (**C**) Staining for ALP activity was performed on P60 entheses. Scale bar: 20 μm. Data, presented as mean ± SD, are representative of 4–6 mice per WT or *Hyp* control group and at least 7 mice for the *VDR^f/f;ScxCreERt–^* and *Vdr^f/f;ScxCreERt+^* groups. One-way ANOVA followed by Fisher’s least significant difference test was used to analyze significance between all genotype groups. *P < 0.05 versus WT; ^#^*P* < 0.05 versus *Hyp*; a indicates *P* < 0.05 versus *Vdr^f/f;ScxCreERt–^*.

Because restoration of 1,25D signaling after enthesopathy has developed does not attenuate BMP and IHH signaling in *Hyp* and *C^–/–^* entheses, the *Vdr* was deleted in Scx^+^ cells starting on P30 to determine if loss of 1,25D action in mature entheses leads to enthesopathy. *Vdr^f/f^* mice were mated to *ScxCreERt* mice, generating *Vdr^f/f;ScxCreERt+^* mice, after which the mice received tamoxifen injections starting on P30 and were sacrificed on P60. Tamoxifen-treated *Vdr^f/f;ScxCreERt+^* and *Vdr^f/f;ScxCreERt–^* control mice have normal serum mineral ions (Figure 10A). Ablation of the *Vdr* in Scx^+^ cells starting on P30, after enthesopathy has developed, does not alter staining for SafO or BMP and IHH markers PTCH and RUNX2 in entheses relative to control entheses. Quantitation for the percentage of positive cells showed that immunoreactivity for p-SMAD1/5/8 was slightly higher in tamoxifen-treated *Vdr^f/f;ScxCreERt+^* entheses versus WT entheses but still significantly lower than that observed in Hyp mice (Figure 10B). Tamoxifen-treated *Vdr^f/f;ScxCreERt–^* control entheses were normal for staining of BMP and IHH markers. ALP activity was significantly higher in both *Vdr^f/f;ScxCreERt–^* and *Vdr^f/f;ScxCreERt+^* entheses compared with WT entheses but still lower than in *Hyp* entheses, as confirmed by quantitation of the percentage of positive cells in entheses (Figure 10C). These studies show that impairing 1,25D action in mature entheses does not lead to enthesopathy.

## Discussion

Our previous investigations demonstrated that the treatment of *Hyp* mice with either 1,25D or FGF23Ab, which also increases 1,25D levels, prior to enthesopathy development prevents enthesopathy (14). Because both treatments attenuated *Hyp* enthesopathy despite dramatic increases in *Fgf23* expression (15), these studies implicate impaired 1,25D action due to the high serum levels of FGF23 in XLH rather than direct effects of increased circulating FGF23 in the pathogenesis of XLH enthesopathy. Our current studies demonstrate that Achilles entheses from all groups of mice lacking 1,25D action, regardless of whether these mice are null for *Fgf23* or *Phex* (*C^–/–^, C^–/–^/Hyp, C^–/–^/F^–/–^, C^–/–^/F^–/–^/Hyp*), have enhanced BMP and IHH signaling and ALP activity, thus confirming that impaired 1,25D signaling as a result of increased serum FGF23 levels in *Hyp* mice leads to enthesopathy.

Deletion of FGF23 in mice resulted in a mild, though nonsignificant, decrease in enthesis BMP and IHH signaling compared with WT, whereas deletion of *Fgf23*in *Hyp* mice restored normal BMP and IHH signaling in *Hyp* entheses, suggesting actions specific to FGF23 or actions downstream of FGF23, such as decreased 1,25D production, regulate enthesopathy development. However, deletion of *Cyp27b1* in *F^–/–^* and *F^–/–^/Hyp* mice (*C^–/–^/F^–/–^ and C^–/–^/F^–/–^/Hyp*) resulted in increased BMP and IHH signaling in entheses, similar to that seen in *Hyp* and *C^–/–^* control mice, thus demonstrating that impaired 1,25D production due to elevated FGF23 levels, not actions specific to FGF23, results in XLH enthesopathy. Deletion of *Phex* in *C^–/–^* and *C^–/–^/F^–/–^* mice (*C^–/–^/Hyp and C^–/–^/F^–/–^/Hyp*) did not lead to further increased immunoreactivity for BMP and IHH target genes or ALP activity in entheses, suggesting PHEX-specific functions are unlikely to play a dominant role in XLH enthesopathy development. Like mice and humans with XLH, mice overexpressing *Fgf23* (32) and humans deficient in *DMP1* (33) or *ENPP1* (34, 35) have high circulating levels of FGF23, decreased 1,25D levels, and enthesopathy, further supporting our results that impaired 1,25D action as a consequence of high circulating levels of FGF23 underlies enthesopathy development in hypophosphatemic rickets.

*C^–/–^* and *C^–/–^/F^–/–^* mice weaned onto a rescue diet have normal mineral ions, yet entheses from these mice develop enthesopathy, indicating that actions specific to 1,25D, independent of its ability to regulate calcium and phosphate homeostasis, suppresses BMP and IHH signaling in entheses. Similar to *C^–/–^* mice, mice lacking the *Vdr* in *Scx*-expressing cells (*VDR^f/f;ScxCre+^* mice) had normal mineral ion homeostasis as well as Achilles entheses with enhanced BMP and IHH signaling. These results demonstrate that 1,25D acts directly on Scx-expressing cells to inhibit BMP and IHH signaling in entheses.

Fibrocartilage enthesis cells descend from *Gdf5*-expressing cells (26). Furthermore, *Gdf5* promotes and regulates chondrocyte hypertrophy and maturation (27). The present studies demonstrate that both *Hyp* and *C^–/–^* entheses have increased expression of *Gdf5*, suggesting that enhanced GDF5 action contributes to the increased BMP and IHH signaling in *Hyp* and *C^–/–^* entheses and, thus, enthesopathy development. Furthermore, 1,25D suppresses GDF5-induced BMP and IHH signaling, including phosphorylation of SMAD1/5, protein expression of SMAD1, and gene expression of *Smad4*, and directly decreases expression of BMP and IHH target genes. These data indicate that the interaction of 1,25D and BMP and IHH signaling is important for enthesis maturation and enthesopathy development. The elevated FGF23 levels in *Hyp* mice lead to impaired 1,25D production. Based on our findings that 1,25D acts on enthesis cells to suppress BMP and IHH signaling, the decrease in VDR expression in *Hyp* entheses likely also contributes to the impaired 1,25D action in *Hyp* entheses, leading to increased BMP and IHH signaling and enthesopathy development.

In support of our data that 1,25D suppresses BMP signaling, 1,25D treatment of rat bone marrow stromal cells or UMR-106 rat osteosarcoma cells decreases *Bmp2* mRNA expression (36). Moreover, bone expression and serum levels of BMP2 are elevated in mice with either global or osteocyte-specific deletion of *Cyp27b1* (*Oct-C^–/–^*) relative to WT mice (37). The elevated BMP2 expression in *C^–/–^* and *Oct-C^–/–^* mice with chronic renal disease was associated with worse extraskeletal mineralization in the aorta and heart compared with WT mice with kidney disease (37), consistent with our hypothesis that the ability of 1,25D action to suppress BMP signaling is protective against ectopic tissue mineralization, like enthesopathy.

*Hyp* mice treated with 1,25D or FGF23Ab (15) starting on P2 have improved skeletal mineralization, pointing to a possibility that increased mineralization, not altered mineral hormones, prevents enthesopathy. However, our data demonstrate a lack of SafO^+^/ALP^+^ HECs in *F^–/–^* and *F^–/–^/Hyp* mice (Figure 2), which both have elevated serum 1,25D levels (7) and decreased bone density and osteomalacia (7). Also, *C^–/–^* mice fed a rescue diet do not have impaired mineralization (38), yet they develop enthesopathy, as evidenced by the enhanced BMP and IHH signaling and ALP activity in *C^–/–^* entheses (Figure 2, Figure 3, Figure 4, and Figure 5). Thus, these results further support a role for 1,25D action in enthesopathy development, independent of its effects on skeletal mineralization.

*Hyp* entheses have enhanced BMP and IHH signaling and ALP activity by P14, which is prevented if increasing 1,25D action with 1,25D or FGF23Ab therapy is initiated by P2 (prior to development of enthesis changes), suggesting XLH enthesopathy develops in the early postnatal period (14). Like *Hyp* mice, *C^–/–^* entheses also develop SafO^+^, ALP^+^ HECs with enhanced BMP and IHH signaling by P14, when this increase in BMP and IHH signaling is attenuated if 1,25D signaling is restored starting on P2, thus demonstrating lack of 1,25D action leads to enhanced BMP and IHH signaling in entheses starting early in postnatal development. Consistent with our murine data that enthesopathy can be prevented if treatment with 1,25D is started early in postnatal development, an observational clinical study demonstrated that a lower incidence of enthesopathy in adults with XLH is associated with continuous therapy with combination phosphate and 1,25D from ages 3 to 18 years, but not with adults with XLH who had periodic or no treatment as children (39). In further support of our results that enthesopathy development is blocked if therapy is initiated early, Herrou et al. (40) showed in a retrospective clinical study that longer duration of conventional therapy with phosphate and active vitamin D analogs like 1,25D during childhood was associated with a lower incidence of enthesopathies in adults with XLH.

In contrast, restoring 1,25D signaling with 1,25D therapy starting at P30 (after enthesopathy has already developed) in either *Hyp* or *C^–/–^* mice was not able to substantially decrease the enhanced BMP and IHH signaling observed in the entheses of these mice, indicating that enhancing 1,25D signaling in mature entheses does not attenuate enthesopathy. Taken together with our data showing that 1,25D prevents enthesopathy in *Hyp* (14) and *C^–/–^* mice with early initiation of therapy, these studies demonstrate that 1,25D prevents differentiation of normal enthesis cells into HECs, but 1,25D is unable to attenuate BMP and IHH signaling after differentiation into HECs has occurred and enthesopathy has developed.

In support of our findings, other studies have reported that treatment of *Hyp* mice with intermittent 1,25D combined with phosphate supplementation after enthesopathy has already developed did not suppress ALP activity in *Hyp* entheses (32, 41). Consistent with the murine data that enthesopathy cannot be reversed (32), an observational cross-sectional study suggested treatment of patients with XLH during adulthood with combination phosphate supplementation and low doses of 1,25D does not affect the number of entheses affected by pathological mineralization (42). It was not clear at what stage in enthesopathy progression treatment was begun. An anti–FGF23 mAb (burosumab) approved for XLH treatment of affected children and adults, is not able to maintain serum 1,25D levels, which decline after initiation of therapy (43–45), similar to 1,25D levels in FGF23Ab-treated mice, which decrease by P75 (14). There are no data available yet on the effect of burosumab on enthesopathy prevention or treatment in those affected with XLH.

Deletion of the *Vdr* in Scx^+^ cells throughout enthesis development results in increased BMP and IHH signaling in entheses (Figure 7), whereas deletion of the *Vdr* in Scx^+^ cells starting at P30 in *Vdr^f/f;ScxCreERt+^* mice (Figure 9) did not lead to enthesopathy. Consistent with 1,25D being able to prevent but not reverse enthesopathy in *Hyp* and *C^–/–^* mice, these results demonstrate that impairing 1,25D action starting early in enthesis development leads to enhanced BMP and IHH signaling in entheses, but impairing 1,25D signaling in mature entheses does not affect BMP and IHH signaling in entheses. These results suggest that 1,25D signaling regulates enthesis cell differentiation early in postnatal development but cannot modify enthesis organization after entheses have matured. We show that there is a progressive decrease in VDR expression as entheses age, regardless of genotype, which may contribute to the resistance of older enthesis cells to 1,25D. Published studies demonstrated that enthesis cells from rats that age from 2 months to 24 months of age have progressively decreased chondrogenic potential and ability to proliferate (46), thus reinforcing our findings that BMP and IHH signaling is less modifiable by 1,25D in entheses as mice age. The present studies demonstrate that although BMP and IHH signaling remains elevated in *Hyp* and *C^–/–^* entheses, expression of BMP and IHH signaling target genes decreases by P60. Despite this relative decrease in BMP and IHH signaling in older entheses, the expression of BMP factors (*Bmp2*, *Bmp4*, and *Bmp6*) is not lower, and the expression of BMPRs 1A and 2 is not changed, compared with P14 entheses. Expression of *Gdf5* and its preferred receptor *Bmp1b* is decreased in P60 versus P14 entheses, indicating this could play a role in the decreased BMP and IHH signaling observed with aging.

Taken together, our results demonstrate that in XLH, impaired 1,25D action due to high serum levels of FGF23, not the direct effects of increased FGF23, underlies the pathogenesis of enthesopathy. 1,25D acts directly on enthesis cells to suppress BMP and IHH signaling and can prevent enthesopathy in *Hyp* and *C^–/–^* mice if 1,25D therapy is initiated early in postnatal development, indicating that 1,25D signaling is protective against enthesopathy development. These findings support the beneficial effects of early, consistent, and optimized 1,25D or FGF23Ab therapy. Enhancing 1,25D signaling early in development may prevent the development of XLH enthesopathy.

## Methods

### Animal studies.

All mice were on a C57BL/6J background. Mice of each genotype were backcrossed at least 8 generations into the C57BL/6J background. All mice were maintained in a virus- and parasite-free barrier facility, exposed to a 12-hour light/dark cycle, and housed with up to 5 mice per cage. To examine the relative roles of 1,25D, FGF23, and PHEX in enthesopathy development, mice lacking *Cyp27b1*, *Fgf23,* or both, with or without the *Hyp* mutation, were analyzed. *Cyp27b1* null mice (*C^–/–^*) lack exon 8, a region coding the heme-binding domain (originally provided by Rene St. Arnaud, Shriners Hospital for Children, Montreal, Quebec, Canada) (19). *Fgf23* null mice (*F^–/–^*) have a knockin of EGFP following the ATG in exon 1 (7) (*Mutant Mouse Resource and Research Center*; catalog 036748-JAX). *C^–/–^* mice were mated to *F^–/–^* mice to generate mice null for both *Cyp27b1* and *Fgf23* (*C^–/–^/F^–/–^*). *Hyp* mice (The Jackson Laboratory; JAX stock catalog 000528) were mated to *C^–/–^*, *F^–/–^*, and *C^–/–^F^–/–^* mice, generating *Hyp* mice null for *Cyp27b1* (*C^–/–^/Hyp*), *Fgf23* (*F^–/–^/Hyp*), or both (*C^–/–^/F^–/–^/Hyp*).

To delete the *Vdr* in Scx-expressing cells, mice carrying exon 4 floxed alleles (originally provided by David Gardner, UCSF, San Francisco, California, USA) (31) were mated to *Scx-Cre* mice (4) (*Vdr^f/f;ScxCre+^*) and *Scx-CreERt* mice (47) (*Vdr^f/f;ScxCreERt+^*) (both strains of mice originally were provided by Ronen Schweitzer, Oregon Health and Science University, Portland, Oregon, USA). Tamoxifen (0.1 mg/g; Sigma) dissolved in sunflower oil was injected i.p. on P30 and P31 into *Vdr^f/f;ScxCreERt+^* mice. The enthesis phenotype of *Vdr^f/f;ScxCre+^* mice and tamoxifen-treated *Vdr^f/f;ScxCreERt+^* mice were compared with that of age- and sex-matched *Vdr^f/f;ScxCre–^* and tamoxifen-treated *Vdr^f/f;ScxCreERt–^* mice, respectively. Age- and sex-matched WT and *Hyp* mice that were littermates of each other served as additional controls.

Equal numbers of male and female mice were included in each group for all analyses. All groups of mice lacking *Cyp27b1* (*C^–/–^, C^–/–^/Hyp, C^–/–^/F^–/–^, C^–/–^/F^–/–^/Hyp*) were fed a gamma-irradiated rescue diet (catalog TD96348; Harlan Teklad) with 2% calcium, 1.25% phosphate, 20% lactose, and 2.2 IU vitamin D/g) starting on day 18 to prevent hypocalcemia and hypophosphatemia (48). All other mice were weaned on day 18 onto house chow (0.8% calcium, 0.6% phosphate; PicoLab 5053).

### Calcitriol treatment.

Male and female *C^–/–^* mice were injected daily with 1,25D (Akorn Inc.) at 175 pg/g/d from P2 to P18 and then 80 pg/g/d after P18. The dose of 1,25D was decreased on P18 when mice were weaned onto the rescue diet to prevent hypercalcemia. Male and female *Hyp* mice were treated with 175 pg/g/d from P30 to P60 (14). Sex- and age-matched WT mice, with *Hyp* or *C^–/–^* littermates, served as the control and received vehicle.

### Serum parameters.

All mice were fasted for 2 hours, after which serum from mice was obtained by cheek bleeding. Serum calcium and phosphate levels were measured colorimetrically using kits from Stanbio and Abcam, respectively.

### Histology.

As previously described (14), the Achilles tendon entheses were dissected from mice, fixed in 10% formalin/PBS, decalcified in 20% EDTA/PBS (pH 8.0), and processed for paraffin sections. For frozen sections, decalcified entheses were immersed in a sucrose gradient and then embedded in OCT and cryosectioned at 5 μm. To perform the SafO stain, deparaffinized enthesis sections were rehydrated, counterstained with 0.02% Fast Green (MilliporeSigma), followed by incubation in 0.1% SafO (MilliporeSigma) (14). To evaluate ALP activity, decalcified, frozen enthesis sections equilibrated to room temperature were immersed in Tris buffer at pH 9.4 for 1.5 hours and then incubated in ALP staining solution (Tris buffer at pH 9.4, fast blue solution, naphthol ASBI phosphate solution, and magnesium chloride) at room temperature (14).

### IHC.

IHC analyses for p-SMAD1/5/8, RUNX2, PTCH (14), and the VDR were performed. Paraffin sections were rehydrated and blocked with TNB (0.1 M Tris-HCL pH 7.5, 0.15 M NaCl, 0.5% blocking reagent [PerkinElmer]). For p-SMAD1/5/8, RUNX2, and VDR IHC, sections were incubated with proteinase K at 37°C. After incubation with primary Ab (anti–p-SMAD1/5/8 Ab, 1:250, MilliporeSigma, catalog AB3848-I; and anti–RUNX2 [D1L7F] Ab 1:500, Cell Signaling Technology, catalog 12556), or anti–VDR (D2K6W) Ab (1:900; Cell Signaling Technology; catalog 12550S), signal detection was performed using biotinylated goat–anti rabbit secondary Ab (Vector Laboratories), followed by amplification with the TSA Kit (Akoya Biosciences) and then incubation with streptavidin conjugated with HRP (PerkinElmer). For PTCH IHC, deparaffinized sections were subjected to antigen retrieval with trypsin, blocked with 10% heat-inactivated FBS in PBS, and incubated with anti–PTCH1 Ab (1:500; Abcam; catalog ab53715). Signal was detected using a goat anti–rabbit HRP Ab (MilliporeSigma).

### Histomorphometry.

The percentages of SafO^+^, ALP^+^, and immunoreactive cells in the entheses were quantitated as previously described (14). The enthesis region was defined on 1 side by the distal border of the calcaneal secondary ossification center and on the other side by the point where the anterior part of the tendon emerges from the articular surface. The ratio of the number of immunoreactive, SafO^+^, or ALP^+^ cells to the total number of cells in the defined enthesis region was calculated. The investigators performing these analyses were blinded to the identities of the samples.

### Isolation of enthesis RNA.

The site at which the P14 or P60 WT, *Hyp*, and *C^–/–^* Achilles tendon insert into the calcaneus was identified under a dissection microscope (Nikon) at ×5 magnification and isolated using a microsurgical knife by slicing through the proximal end of the tendon and the region adjacent to the distal portion of the calcaneus (14). The enthesis tissue was homogenized in Trizol (Thermo Scientific Fisher). Total RNA was precipitated using 100% alcohol, purified using the Quick*-*RNA Tissue/Insect Kit (Zymo Research).

### In vitro chondrocyte studies.

Primary chondrocytes were isolated from P2 WT murine ribs and cultured as previously described (14, 48). Chondrocytes were cultured for 48 hours and then serum restricted (0.5% FBS). For Western blot analyses, chondrocytes were pretreated for 0.5, 1, 4, or 18 hours with 1 × 10^–8^ M 1,25D prior to exposure to recombinant human GDF5 (rhGDF5) (100 ng/mL; R&D Systems) for 30 minutes. For gene expression analyses, chondrocytes were pretreated for 18 hours with 1 × 10^–8^ M 1,25D prior to exposure to rhGDF5 (200 ng/mL) for 4 hours. Chondrocytes were harvested for protein or RNA (PureLink RNA mini kit; Thermo Fisher Scientific) after 72 hours in culture.

### Western blot analyses.

Protein concentration was quantitated using the BCA protein assay (Pierce) and 7.5 μg of protein was subjected to Western blot analysis. Membranes were blocked in EveryBlot blocking buffer (Bio-Rad; catalog 12010020) and then incubated with primary Abs against p-SMAD1/5/9 (1:750; Cell Signaling Technology; catalog 13820S), SMAD1 (1:1000; Cell Signaling Technology; catalog 6944S), or Actin (1:1000; Cell Signaling Technology; catalog 4970S), followed by incubation in HRP-conjugated secondary Abs (Cell Signaling Technology; catalog 7074S). Signals were detected with ECL Plus (Amersham Biosciences).

### Real-time quantitative PCR analyses.

RNA was reverse transcribed with SuperScript II (Roche). Real-time quantitative PCR was performed using the QuantiTect SYBR Green RT-PCR kit (Qiagen) on the Light Cycler 480 II (Roche). Gene expression was quantitated relative to β-actin and then normalized to the corresponding WT entheses of the same age isolated on the same day (for enthesis RNA) or control-treated chondrocytes (for primary chondrocyte RNA) for each sample, using the methods of Livak and Schmittgen (49).

### Statistics.

All data shown are reported as mean ± SD. One-way ANOVA followed by Fisher’s least significant difference test was used to analyze significance between all control and genotype or treatment groups (15, 50). Significance was defined as *P* < 0.05. Because male and female mice have uniform phenotypes within each mouse genotype, analyses are performed with each group including equal numbers of mice of both sexes.

### Study approval.

All animal studies were approved by the IACUC at Brigham and Women’s Hospital (Boston, MA). This study was performed in accordance with the recommendations in the NIH’s Guide for Care and Use of Laboratory Animals.

### Data availability.

All data presented in this manuscript and in the Supplemental Material are available upon request from the corresponding author.

## Author contributions

ESL designed the project. RR, JTB, MS, SV, and ESL contributed to mouse colony management. All authors contributed to molecular biology experiments. Histological analyses were conducted by RR, JTB, MS, SV, SA, and ESL; histomorphometric analyses were conducted by RR, SA, and ESL. SA and ESL prepared the manuscript. SJ contributed to the primary chondrocyte experiments.

## Supplementary Material

Supplemental data

Supporting data values

## Version 1 07/25/2023

In-Press Preview

## Version 2 09/08/2023

Electronic publication
