# Supplementary material for: Impaired 1,25-dihydroxyvitamin D3 action underlies enthesopathy development in the Hyp mouse model of X-linked hypophosphatemia
Source: JCI Insight. 2023 Sep 8;8(17):e163259. doi: 10.1172/jci.insight.163259 (PMC10544216; doi:10.1172/jci.insight.163259)
Supplement: Supplemental data [file jciinsight-8-163259-s197.pdf]

Supplemental Figure 1

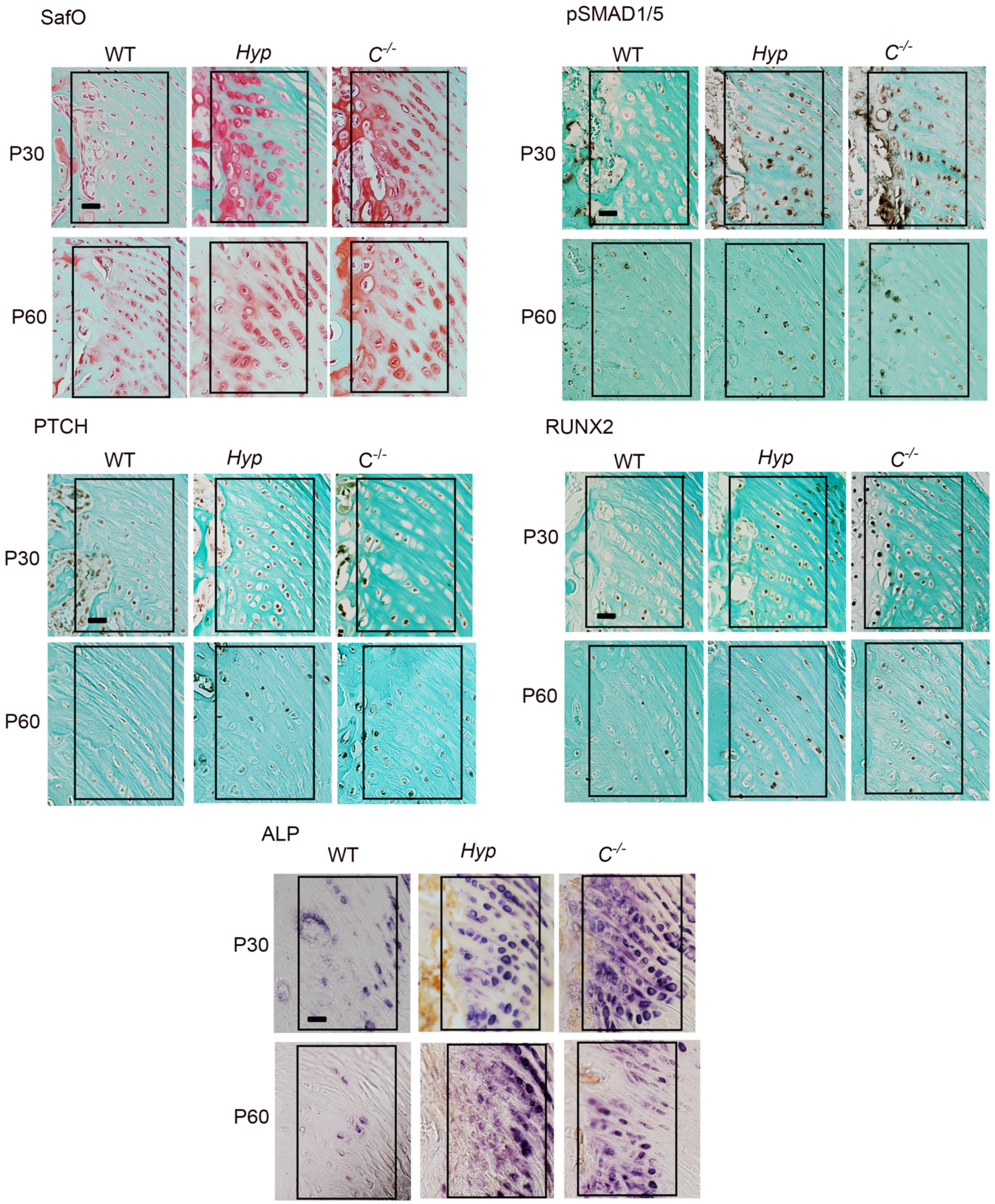

Supplemental Figure 1: Enhanced BMP/IHH signaling observed in *Hyp*, and *C*$^{-/-}$ entheses decrease by P60. Staining for Safranin O (SafO), immunohistochemistry performed for p-SMAD1/5/8, PTCH, and RUNX2, and staining for alkaline phosphatase activity (ALP) P30 and P60 WT, *Hyp*, and *C*$^{-/-}$ entheses**.** Scale bar = 20 µm. Data are representative of at least 6 mice per age/genotype group. In all representative pictures, the enthesis region is outlined with a black box.

Supplemental Figure 2

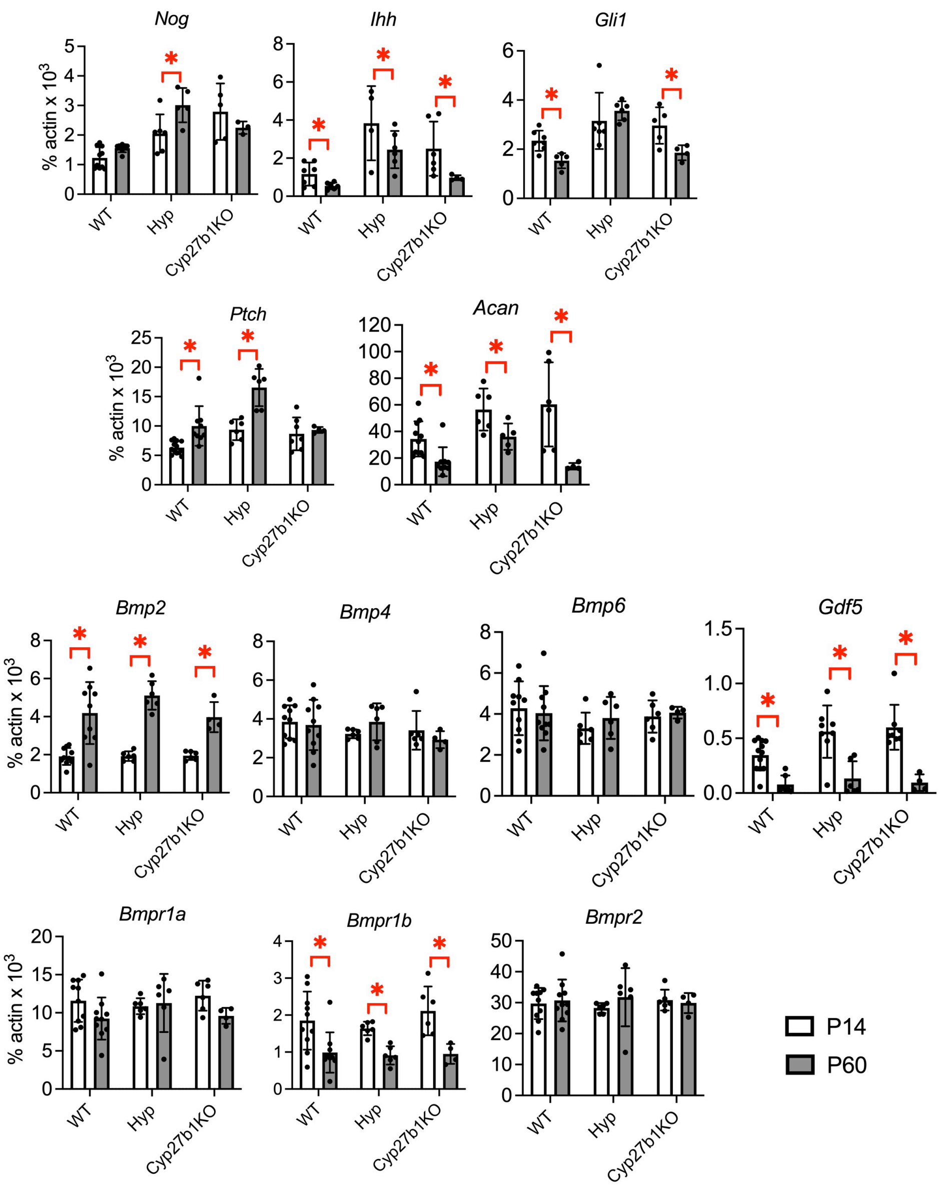

Supplemental Figure 2: mRNA expression of BMP/IHH target genes in entheses decrease by P60. mRNA expression of BMP and IHH signaling genes, *Acan*, and *Scx* in P14 and P60 WT, *Hyp*, and $C^{-/-}$ entheses. Gene expression is normalized to that of actin. Data, presented as mean±SD, are representative of at least 6 mice per genotype in P14 entheses and 4-6 mice per genotype in P60 entheses. Two-way ANOVA followed by Fisher's LSD test was used to analyze significance between all genotype groups. * =p value <0.05 P14 vs P60 for each genotype. The same data is represented as fold change in gene expression relative to WT in Figure 5. Significant changes in expression of genes between WT, *Hyp*, and *Cyp27b1KO* mice at each time point are shown in Figure 5.
